# De Novo Genes

**DOI:** 10.1146/annurev-genet-111523-102413

**Published:** 2024-11-14

**Authors:** Li Zhao, Nicolas Svetec, David J. Begun

**Affiliations:** 1 Laboratory of Evolutionary Genetics and Genomics, The Rockefeller University, New York, NY, USA; 2 Department of Evolution and Ecology, University of California, Davis, California, USA

**Keywords:** gene gain, gene loss, adaptive evolution, regulation of expression, de novo proteins, small ORFs

## Abstract

Although the majority of annotated new genes in a given genome appear to have arisen from duplication-related mechanisms, recent studies have shown that genes can also originate de novo from ancestrally nongenic sequences. Investigating de novo–originated genes offers rich opportunities to understand the origin and functions of new genes, their regulatory mechanisms, and the associated evolutionary processes. Such studies have uncovered unexpected and intriguing facets of gene origination, offering novel perspectives on the complexity of the genome and gene evolution. In this review, we provide an overview of the research progress in this field, highlight recent advancements, identify key technical and conceptual challenges, and underscore critical questions that remain to be addressed.

## INTRODUCTION

Questions related to the genetics and evolution of novelty are old problems in evolutionary biology. Most rhetorical framing of these issues focuses on phenotypes and emphasizes functional novelty rather than the de novo origin of traits. This is a proper framing, as the conventional view is that all evolved traits, including those that appear to be novel, result from the modification of ancestral traits by natural selection. Indeed, there are many spectacular examples of functional novelty associated with phenotypes that have become so dramatically modified from their ancestral states that the evolutionary origins of the derived traits were challenging to infer. Among the most famous examples is the origin of the vertebrate inner ear, which derives from the highly modified jawbones of ancestors (44, 63). More recently discovered examples of novel phenotypes resulting from the redeployment of ancient phenotypes to new developmental contexts include the origin of feathers (22); the repeated evolution of lens crystallins from soluble enzymes (129); and the evolution of wing patterns (17, 95), body coloration (135), and male reproductive phenotypes (46, 114), among others.

What role do evolutionarily novel genes play in the evolution of new traits? Evidence supporting their importance includes the pervasiveness of gene duplications of many types (25, 82, 83, 101), with some deeply investigated examples associated with novel functions (21, 25, 31, 33, 53, 132). However, the relative importance of the various underlying population genetic mechanisms for the spread and fixation of duplicates remains a subject of active investigation (52, 170). While gene duplications are often associated with novel phenotypes (e.g., gene expression, biochemical activity, and subcellular localization) likely resulting from novel regulatory or protein variants (25, 31, 33, 72, 82), these novel genes are fully or partially derived from preexisting genes. Can any complex biological object be truly novel in an evolutionary sense? Perhaps. Indeed, novel genes may have played a pivotal role in the early stages of life, particularly when the genetic pool was limited. It is possible that early RNA molecules or DNA sequences were formed by random associations, arising de novo in this primitive context.

**Figure 1:**
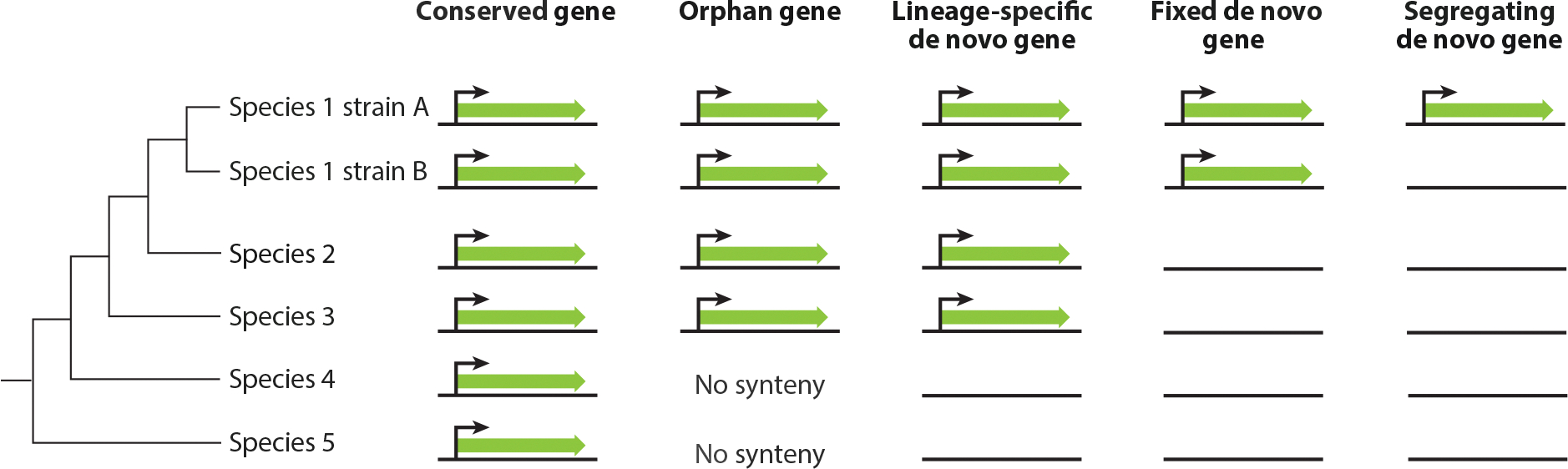
Definitions of gene classes. Black arrows indicate the presence of transcription. Long green arrows represent the transcript, while black lines represent syntenic DNA sequences.

The last few decades have seen the emergence of an alternative mechanism to duplication as the sole source of novel genes. This mechanism, referred to as de novo gene evolution, posits that genes may occasionally emerge from ancestrally nongenic DNA (Figure 1; for a more nuanced and general definition of de novo genes, see the section titled Defining De Novo Genes). While these genes have unusual evolutionary histories, they may provide our best example of how complex functions can derive from nonfunctional ancestry; indeed, they may present one of the evolutionary examples of something from nothing. Their contributions to genome evolution and organismal biology remain largely unclear but of great interest. In this article, we summarize the history of the field, its current state, and outstanding questions.

## DEFINING DE NOVO GENES

**Figure 2:**
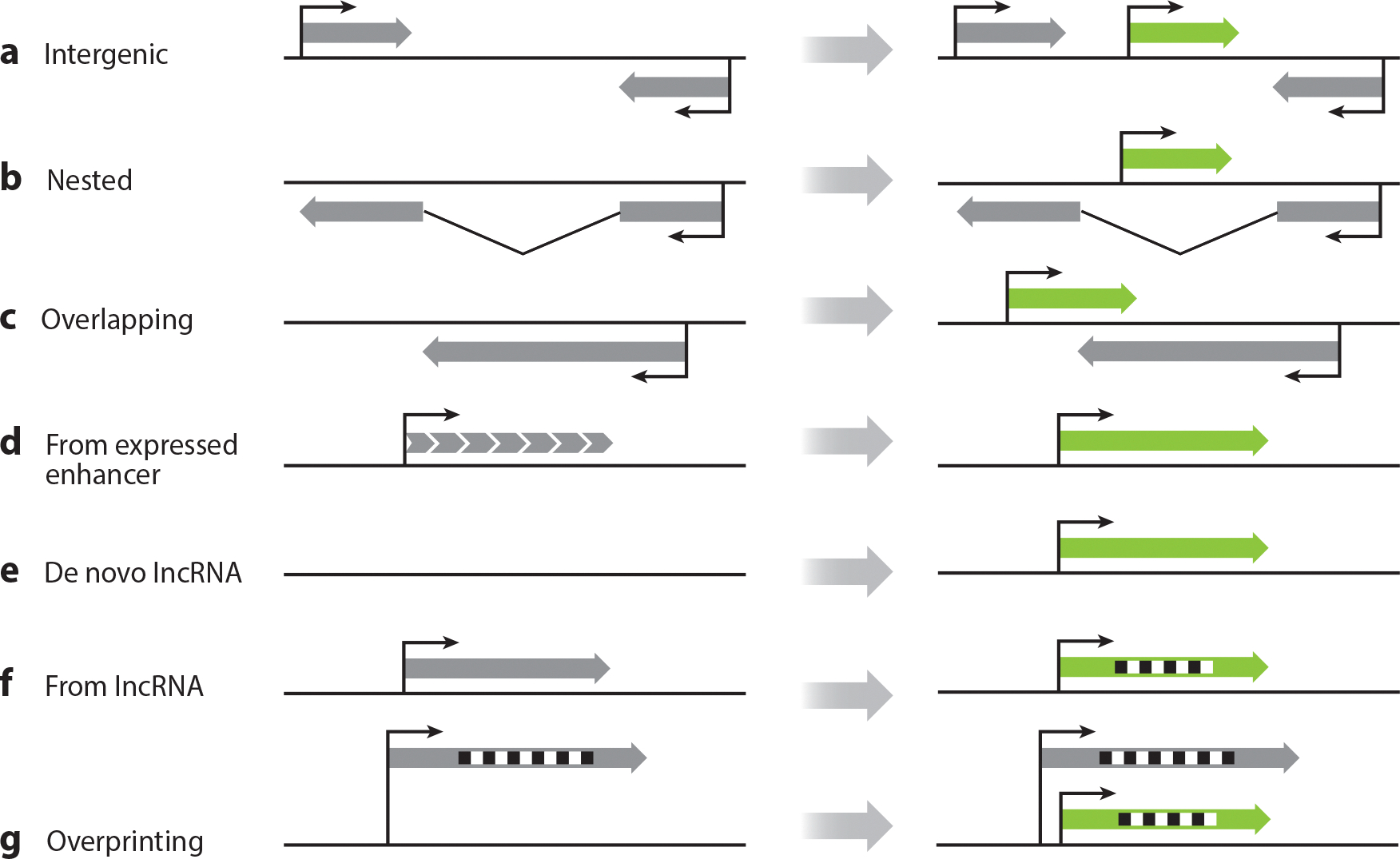
Locations and mechanisms of de novo transcript and de novo gene origination. A de novo transcript (*a*) can originate in intergenic regions, (*b*) can be nested in intronic regions, or (*c*) may overlap and be antisense to an existing transcript. (*d*) An expressed enhancer can serve as a starting point for the emergence of a de novo transcript. (*e*) A de novo lncRNA can be formed from an intergenic sequence. (*f*) A de novo protein can also arise from an existing lncRNA. (*g*) Overprinting involves the emergence of a transcript that overlaps in the same sense with an existing gene but in a different ORF. ORFs are represented by black and white squares. If an ORF is shown in the scheme, it means that ORF recruitment is an essential step in that process. The gray arrows represent canonical transcripts, while the green arrows represent the de novo transcripts. Abbreviations: lncRNA, long noncoding RNA; ORF, open reading frame.

De novo genes are usually defined as genes that originate from ancestrally nongenic sequences, though as we discuss below, this simple definition may not cover all origination mechanisms. How de novo genes are defined is important not only conceptually but also because these definitions are central to the way such genes are identified and analyzed. Nevertheless, there is wide variation in the definitions used. We raise this issue not to prescribe a definition that all investigators should use, or even to suggest that our current understanding of the scientific issues would allow such a definition, but to make the reader aware of this issue when evaluating the literature. For example, if a gene is a DNA sequence producing an RNA, then a de novo gene produces an RNA that is not homologous to an ancestral RNA. Two physical properties of de novo genes follow from this definition. First, they may be protein coding or noncoding. Second, they may originate from any sequence that ancestrally did not produce an orthologous RNA. Thus, while ancestral intergenic or intronic sequences producing novel transcripts would fall under this definition (Figure 2a,b), so would a novel antisense RNA that shares sequence space with an ancestral gene (Figure 2c). In certain cases, enhancers can acquire promoter-like properties and generate a de novo transcript (Figure 2d). An ancestrally nongenic sequence can produce a functional de novo long noncoding RNA (lncRNA) (Figure 2e). Alternatively, an ancestral, functional noncoding gene may evolve a derived protein-coding function (Figure 2f), which would represent a remarkably interesting gain of function (118, 119, 167, 171). However, whether such cases, similar to overprinting (Figure 2g), should be defined as de novo genes is debatable since they originate from preexisting genes. Some investigations start from the proposition that de novo gene candidates, by definition, carry open reading frames (ORFs), making the retention or acquisition of ORFs a key step in de novo gene origination. However, such approaches leave aside a large potential class of de novo genes, such as de novo lncRNAs. MicroRNAs and other types of functional small RNAs may also evolve de novo (84, 149, 152). That said, knowledge about noncoding de novo genes is limited, in part owing to pervasive transcription in many eukaryotic genomes. Therefore, for this review, we focus primarily on protein-coding de novo genes and will not systematically review noncoding de novo genes.

Compared to conserved genes, de novo genes are frequently restricted to a species or lineage (Figure 1), which is similar to orphan genes. In contrast to most genes, de novo genes are often polymorphically expressed in populations (117, 172), consistent with their young age and relatively short persistence times. Fixed de novo genes are those expressed in all individuals studied (Figure 1).

The term protogenes has been occasionally used interchangeably with de novo genes. However, protogenes are more difficult to define. Originally, the term protogene was used in the following sense by Siepel (127, p. 1694): “to produce a protogene that was sufficiently useful for selection to take hold,” which implied a beneficial—perhaps polymorphic—genic novelty. The term was later used to refer to a sequence producing a transcript or protein that is not defined as a gene (18). If polymorphic protogenes (144) are visible to natural selection, then they are segregating de novo genes, which is in line with Siepel’s original use of the term (127). If they are strictly neutral, they can be considered transcriptional or translational noise caused by pervasive transcription and translation (18, 119). How protogenes may serve as intermediate raw material for selection was reviewed previously (148) and is not specifically reviewed here.

## A BRIEF HISTORY OF THE FIELD

The possibility that protein-coding genes could evolve de novo was first discussed by Stephens (130, 139), who pioneered the idea that gene duplication divergence is an important mechanism for functional divergence and pointed out the possibility of de novo gene origin by stating, “Present knowledge is quite inadequate to determine whether it is possible for new genetic loci to arise de novo, or, in fact, to test its occurrence if the possibility existed” (130, p. 249). Two decades later, Jacob (60) considered this idea and then discarded the point in the same sentence. Stephens’s argument emphasized the possibility of a de novo origin, but there were no tools to address it in the 1950s, while Jacob’s argument was that the probability of a random sequence of amino acids having a function was so small that it could be ignored (60). In fact, no real investigation of the possibility that de novo genes could evolve in natural populations occurred until the early 2000s. The events leading to their discovery started with the realization from early genomics analyses that not all genomes shared the same set of genes. Some genes, nicknamed orphans (or ORFans), were present in a focal species but not in all other members of the clade to which it belonged (35, 37, 64, 139). At least four possible explanations for such observations were proposed: incomplete assemblies, gene loss, extreme sequence divergence leading to obscured homology, and horizontal gene transfer.

The first investigation of de novo gene origination as an alternative hypothesis to explain orphans (9) emerged from earlier investigations of *Drosophila* seminal fluid protein gene turnover (8, 151), which had supported gene gain and/or loss over time. Seminal fluid protein genes are small and frequently lacking functional domains, suggesting the possibility that they could emerge de novo from intergenic sequences, since vast stretches of intergenic sequences are riddled with latent ORFs. This idea was investigated using partial complementary DNA (cDNA) sequences, genome assemblies, and gene expression analysis of three closely related *Drosophila* species, which led to the discovery of small accessory gland–expressed transcripts, many of which carried ORFs with predicted signal sequences, for which no expression or homologous ORFs could be detected in the orthologous regions of closely related species. It was suggested that these genes arose from the expression of these latent ORFs. A computational analysis of the *Drosophila melanogaster* intronic and intergenic sequence revealed very large numbers of single-exon ORFs carrying strongly predicted signal sequences, suggesting that the raw sequence material for the emergence of small, secreted proteins from noncoding DNA was plentiful (9). A whole-genome approach followed, using gene annotation from closely related *Drosophila* species, with *D. melanogaster* as the focal species, and follow-up molecular biology experiments to identify putative lineage-specific de novo genes (77); this article is often regarded as the first explicit de novo gene identification paper in the literature. An interesting finding from that analysis was that de novo genes appear to be frequently testis-biased, a trend supported by later work on *Drosophila* and other species (29, 117, 172, 175). Starting in 2008, other investigations of de novo genes started to appear with a focus on model systems such as rodents (56, 165), fruit flies (55, 113, 175), yeast (16, 18, 79, 146), humans and other primates (68, 78, 167), plants (36, 136, 171), fish (176), viruses (106, 120), and nematodes (109).

## IDENTIFYING DE NOVO GENES

Many investigations of de novo gene origination use existing genome annotations and BLAST analysis to identify genes present in a focal species but apparently absent from related species (18, 77, 97). In principle, any single observation of this configuration could be explained by multiple losses of an ancestral gene. However, this is not parsimonious as a general explanation and becomes implausible as the number of related species exhibiting gene absence increases. Some, but not all, de novo gene studies include syntenic analysis to ensure that the orthologous region harboring a candidate de novo gene is present in all species assemblies (19, 93, 156) and that the apparent absence of a gene cannot be explained by extensive sequence divergence in homologous genes that return nonsignificant BLAST results (155). Variations in annotation quality and completeness of genome assemblies are key factors affecting the reliability of such approaches (68, 77, 127). Incorporating synteny information helps reduce artifacts (16, 77, 145). Recently developed whole-genome alignment tools, such as the progressive Cactus aligner that uses both genic and conserved nongenic sequences for synteny alignments, also help improve accuracy (107). Generally, it is more straightforward to identify young de novo genes that only exist in one or a few species, but it is challenging to confirm ancient de novo genes due to extensive sequence divergence and/or difficult alignment.

Other investigations start from the proposition that de novo genes tend to be short, lowly expressed, and absent from some or most species, making them much less likely than ancient genes to be annotated. These investigations typically use raw transcriptome data alongside existing annotations to identify unannotated, focal lineage–specific genes that are absent from related species (12, 172). Thus, a key piece of evidence supporting de novo gene origination in a focal species would be a lack of transcription from the orthologous region in related species. However, what does it mean to say that there is no transcription? What level of transcription would be likely to be indicative of low-level noise rather than a regulated phenomenon, and what expression level in the focal species is evidence of potential biological function? There are no generally accepted answers to these questions because the phenotypic effects of low-level transcription are poorly understood and may vary across cell types and transcripts (99). In the absence of general principles of the biological significance of low-level transcription, a commonly used strategy is to impose a maximum transcript abundance below which a sequence is classified as untranscribed in outgroups and above which the sequence is classified as transcribed in the focal species [e.g., transcripts per million (TPM) > 1 or > 2], though analyses of background transcription of intergenic regions have also been used (23, 172). Alternatively, some studies have used the presence of a nonfocal species homologous and syntenically located transcript as evidence of gene presence regardless of expression level. Finally, statistical approaches to categorize genes as expressed have been proposed, though it is unclear how general or useful these approaches will be (140). Deep single-cell RNA-sequencing (RNA-seq) data may be useful for identifying genes with important functions that are nevertheless expressed in a few cell types. The current sequencing depth of single-cell RNA-seq is not yet sufficient for identification purposes but can assist in confirming the expression levels and dynamics of cell type–biased candidates (160, 161).

Note that the spurious transcription model of de novo gene origination (9, 77) predicts that, all else being equal, de novo gene birth rates would be greater for intergenic regions associated with higher basal levels of spurious transcription. However, if basal rates of spurious transcription are phylogenetically correlated (99), then orthologous regions may be more likely to be misclassified as expressed in nonfocal species. Much more information about the general relationship between gene expression and biological function, especially for lowly expressed genes, will be required to understand the power and biases of all currently used methods of de novo gene discovery.

For all de novo gene identification approaches, variation in data quality across species is a key factor in interpreting results, especially if the focal species has much better assemblies and other genomic data compared to the nonfocal species. For example, much greater investment has been made in fruit fly (*D. melanogaster*), mouse (*Mus musculus*), baker’s yeast (*Saccharomyces cerevisiae*), and human (*Homo sapiens*) annotations than in those of related species. Regardless of the criteria used for identifying putative species-specific de novo genes in model systems, this situation preordains that many false positives will result and that the number of candidates may decline as the resources associated with related species improve. On the other hand, deeper sequencing at the tissue, cell-type, or single-cell levels in future studies might identify more tissue-biased or conditionally expressed de novo genes.

## ROLES OF NATURAL SELECTION AND GENETIC DRIFT IN DE NOVO GENE EVOLUTION

### Comparative Approaches

Several methods have been developed to infer the history of natural selection on genes (Table 1), each with strengths and weaknesses. However, the role of selection in de novo gene evolution is still poorly understood. One reason for this is that several commonly used tests for inferring purifying or directional selection, such as ratio of nonsynonymous to synonymous substitutions (dN/dS) estimates (47) and McDonald–Kreitman tests (39, 90), rely on relatively long protein-coding genes found in multiple species. Many de novo genes are short, taxonomically restricted, and have dubious protein-coding status. The methods require a substantial number of substitutions to reach statistical significance, reducing their value for the analysis of short sequences. Nevertheless, for older de novo genes harboring long ORFs, there is evidence of purifying selection removing new amino acid mutations (51, 92, 150). To the extent that the curated list of de novo gene candidates is high quality, such observations are incontrovertible evidence of de novo gene function. In general, it is important to remember that many ancient, functional genes have dN/dS ≈ 1; thus, individual gene estimates of dN/dS ≈ 1 do not demonstrate that a gene is free of natural selection, though it is consistent with that interpretation. More generally, the strong tendency in *Drosophila* toward deletion of nonfunctional DNA (108) implies that ancient *Drosophila* de novo genes have been retained by natural selection, regardless of whether there is empirical evidence of function. Global comparisons of evolutionary properties of de novo genes should use comparison sets of ancient genes with similar characteristics, such as short length and low expression, to understand the extent to which de novo gene properties or signatures of selection deviate from those of ancient genes. Furthermore, population genetic or comparative genomic inferences about the influence of selection on individual genes should be interpreted carefully for primarily clonal organisms, as the clone rather than any individual gene is the entity on which selection acts.

### Population Genetic Approaches

Comparative approaches using divergence to investigate the possible role of selection in sequence evolution are challenging to use for very young de novo genes and impossible to apply to species-specific genes, where divergence is meaningless. Nevertheless, population genetic variation can potentially provide insights. For example, analysis of segregating de novo gene candidates in *D. melanogaster* provided evidence of hitchhiking effects (172). While such evidence does not prove that the de novo genes are the targets of selection, this is the most parsimonious interpretation. Correlations between the properties of segregating de novo genes and their population frequencies may provide additional information on selective effects (Table 1). For example, under the hypothesis that de novo genes are deleterious, one would expect lower-frequency genes to be longer and more highly expressed, as those properties would be associated with greater negative fitness effects. Alternatively, under the hypothesis that segregating de novo genes are free of selection, the properties of de novo genes in different frequency classes should be the same. In contrast to these predictions, segregating *Drosophila* de novo genes that are expressed in more genotypes are longer and more highly expressed than those expressed in fewer genotypes, which is suggestive of an effect of positive selection on de novo gene spread (29, 172). Identifying and investigating the population genetics and evolution of genetic variants responsible for de novo gene expression will enable much more incisive population genetic analyses of segregating or recently fixed de novo genes.

An alternative approach for investigating the role of selection on de novo genes found in only one species is based on the idea that for a protein-coding de novo gene the presence of functional constraint predicts that nonsynonymous site heterozygosity (pN) should be lower than synonymous site heterozygosity (pS). Some studies have found a general trend toward pN/pS < 1 for species-specific genes (55, 92, 146), whereas others have observed pN/pS ≈ 1 (119, 150). However, as noted above for dN/dS ratios, pN/pS ≈ 1 for short genes should not be taken as strong evidence for the absence of selection (71). Moreover, pN/pS ratios >1 are also consistent with the presence of deleterious amino acid variation, stronger selection on synonymous sites than nonsynonymous sites, or diversifying selection, all of which support the inference of function.

### Population Genetic Effects on De Novo Gene Dynamics

The birth and subsequent spread of young de novo genes may be affected by many processes that are directly or indirectly related to population genetics. For example, organisms with smaller effective population size or reduced recombination may carry more noncoding DNA (20, 48) and harbor more typically deleterious variants associated with spurious transcription (153), leading to greater de novo gene birth rates compared to species with larger populations and more recombination. However, selection may be less effective in fixing slightly beneficial de novo genes in such species. How these factors, as well as unique biological or ecological attributes of different species, organs, and cell types, influence de novo gene birth and spread is an important area for future investigation. There are hints that local adaptation may play a role in the dynamics of young de novo genes (29, 172), but this too is an area about which little is known.

## REGULATION OF DE NOVO GENES

Transcriptional activation is a key step in de novo gene origination. Without transcription, a DNA sequence cannot be functional as a gene—whether protein-coding or noncoding—nor can it be subject to natural selection as a potential gene product. De novo promoters (168) and enhancers (43, 138) from random DNA are easy to generate, as are promoters (169) and enhancers (38) in various species. Consequently, de novo genes can be expressed dynamically and with a specific spatiotemporal expression pattern even at the cell-type level (160).

**Figure 3:**
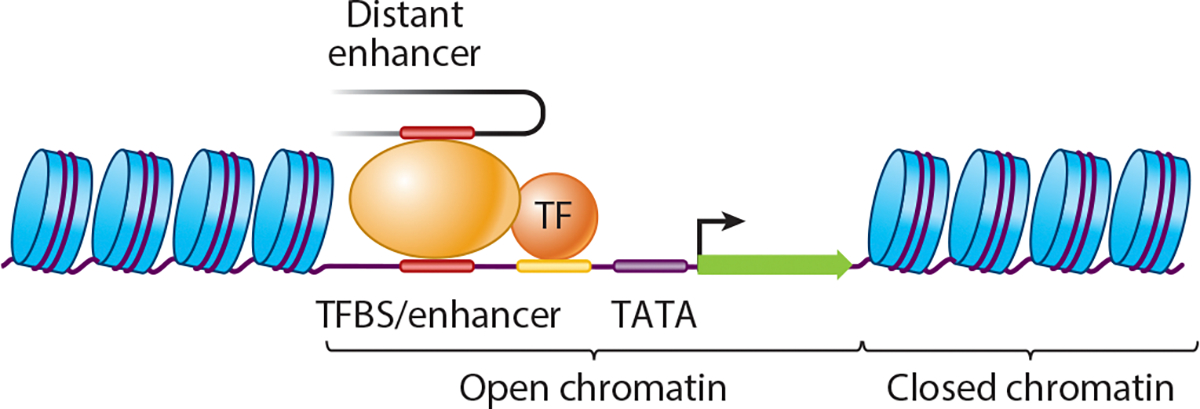
Factors involved in the gain of transcription. An untranscribed DNA region typically requires chromatin remodeling to expose potential TFBSs. The DNA sequences may acquire mutations that increase the binding of TFBSs and develop promoter-like motifs and a TATA box. Additionally, TF binding can also be regulated by distant enhancers, highlighting the importance of the 3D genome structure in gene expression. Abbreviations: 3D, three-dimensional; TF, transcription factor; TFBS, transcription factor–binding site.

Some de novo genes are regulated in *cis*, either by novel promoters or by co-opting preexisting enhancers or promoters in the nearby regions (89, 172), such as the use of bidirectional promoters (163). Consequently, the expression patterns of some de novo genes have a higher correlation with nearby genes than what random simulations suggest. This implies that they are either reusing *cis*-regulatory sequences or chromatin contexts or that the de novo genes and their neighboring genes are regulated by an overlapping set of transcription factors. Some de novo genes might be influenced by the bidirectional promoters or enhancers of other genes, and, in return, indirectly regulate other genes through the competition of promoters and enhancers. It is also intriguing to hypothesize whether the shared or overlapping regulatory machinery of de novo genes and other conserved genes impacts their fitness effect, whether positively or negatively, and how this influences the evolutionary trajectory of de novo genes. This mechanism might be more relevant to some de novo genes located near or within annotated conserved genes (Figure 2a–c) or genes that are regulated by a similar set of transcription factors (162). The observation that some antisense de novo genes interact with their host genes at both transcriptional and protein levels may support such a hypothesis (79). Another important question pertains to the role of three-dimensional (3D) genome organization (76) and the role that distant enhancers may play in de novo gene regulation (Figure 3), which is largely unstudied.

One largely unexplored question concerns the proper maintenance and degradation of transcripts of de novo genes. The nonsense-mediated messenger RNA decay (NMD) system serves as a surveillance mechanism for scrutinizing messenger RNA (mRNA) quality (86) and ensuring translational accuracy. NMD primarily targets protein-coding mRNAs but sometimes also binds to lncRNAs (137, 177). It is fascinating to consider the impact of NMD on gene origination steps: It could reduce the half-life of de novo transcripts and decrease the probability of accumulating unwanted or deleterious de novo proteins. Therefore, new genes emerging with NMD may be less likely to be retained but also less likely to produce strongly deleterious effects. Single-exon genes are less likely to be targeted by NMD due to the absence of splicing, and key mechanisms of NMD involve the recognition of exon–exon junctions (86). Thus, NMD might be less frequently encountered in shorter or single-exon novel genes than in longer, multi-exon ones.

mRNA polyadenylation is a crucial process involving the cleavage of nascent mRNAs and the addition of the poly(A) tail. De novo genes, including segregating ones, can recruit polyadenylation sites (172). In fact, large-scale datasets have revealed numerous polyadenylation sites across genomes (123), many of which are not directly linked to annotated genes. Some of these novel polyadenylation sites might serve as alternative termination sites for different isoforms of conserved genes. Others, particularly those that are distant from conserved genes and are tissue-specific, might be utilized for novel genes. While classical polyadenylation sites such as AAUAAA are abundant in genomes, it is intriguing to consider whether, in the early steps of gene origination, the transcriptional machinery produces multiple isoforms using different polyadenylation sites, thereby offering diverse products for tinkering by natural selection.

## FUNCTIONS OF DE NOVO GENES

**Figure 4:**
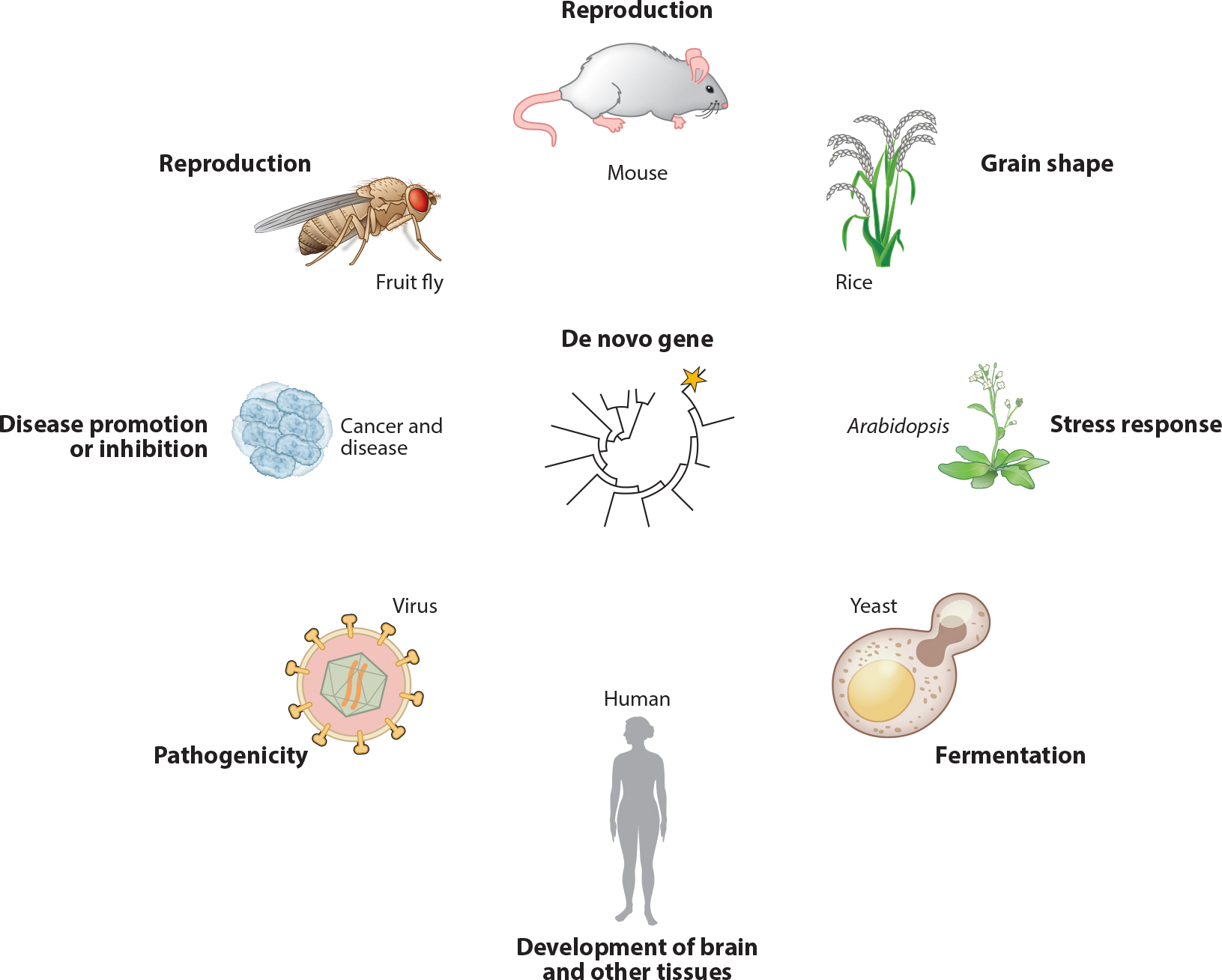
De novo genes perform various functions across different taxa. Examples are from fruit flies (164), mice (56), rice (24), *Arabidopsis* (136), yeast (79, 80), humans (1), and viruses (120), as well as cancer and disease cell types (133, 134).

Identifying de novo gene functions and fitness effects is challenging, partly due to their overall smaller fitness effects compared to conserved genes. Similar to conserved genes of unknown function, the functions of any gene are difficult to study directly without prior knowledge of functional domain or homologous gene function. Despite these challenges, several studies have shed light on de novo gene function, which can be broadly categorized into reproduction-related and nonreproductive functions (Figure 4). In this section, we focus exclusively on the functional studies of naturally occurring de novo genes, not random synthetic proteins.

Systematic research on de novo genes uncovers a distinct expression pattern, predominantly in reproductive tissues such as testes (77) and accessory glands (9, 29) in *Drosophila* and primates (117, 167), as well as in plant pollen (30). In mice, a de novo RNA gene is linked to enhanced reproductive fitness (56). Additionally, a female-expressed gene in mice is indicated in some level of reproductive success (165). In *Drosophila*, some de novo genes are associated with male reproduction (26, 113, 164). Together, these illustrate the diverse functional spectrum of these genes, although concerns about imperfect methodology such as RNA interference (RNAi) (50) in fruit flies have questioned the reliability of some of the findings (164). In *Arabidopsis*, de novo genes play a role in stress response and flowering transition (136). A de novo gene in rice contributes to grain shape differences (24). Two ancient, putative de novo protein-coding genes in fruit flies are essential for male reproduction, highlighting their crucial role in specific biological functions (51, 74, 115). Their de novo origins remain unconfirmed due to difficulties in identifying outgroup homologous sequences.

Nonreproductive examples include the human-specific de novo gene *ESRG*, partly derived from an endogenous retrovirus, involved in maintaining pluripotency in human stem cells (173), a presumed function in other primates. Another case involves *NCYM* (or *MYCNOS*), the anti-sense gene of *MYCN*, serving a specific function of inhibiting *GSK3β* and stabilizing *MYCN* in human neuroblastomas (133, 134). A few de novo genes, such as a lineage-specific ORF of *ENSG00000205704*, might function in brain development (1, 78). In simpler organisms like yeast, one of the best-studied genes is *MDF1*, whose de novo antisense expression functions in inhibiting mating and promoting growth (79, 80). Other studies indicate a subset of segregating de novo genes or protogenes that encode proteins that localize to endoplasmic reticulum (ER) membranes and may have ER-related functions (144).

Studying the functions of young de novo genes presents challenges, partly due to the complexity of natural selection and partly because even variants with small effects can become fixed under selection. Laboratory experiments, including knockout experiments, often lack the power to detect genes with minor or condition-specific fitness effects. The absence of knockout phenotypes could be due to low fitness impact of new genes, oversimplified phenotypic assays, functional redundancy and compensation after knockout, or a lack of functions for new genes. A compromise approach involves studying knockout individuals’ expression levels, assuming that changes in the expression network ultimately affect downstream phenotypes. Several studies support this research direction (33, 165, 166).

De novo genes may arise and fix in populations due to their potential participation in genomic conflicts. Selfish elements in some taxa, such as transposable elements (27), can reach fixation despite being mildly deleterious. Some rapidly evolving duplicated genes have evolved in meiotic drive systems (40, 100), which may influence gene frequency and genome architecture (122). Because genomic conflicts are a chronic yet dynamic source of natural selection (81), it seems possible that the spread of de novo genes may sometimes be the result of selection for suppressors of meiotic drive or transposable element proliferation. Because these genomic conflict systems tend to evolve very quickly, the rapid turnover and short half-life of many de novo genes might reflect the rapidly changing selective forces related to these ongoing conflict dynamics. This phenomenon might also explain the high abundance of testis-expressed de novo genes. Whether similar de novo gene dynamics occur in the female germline is still unclear.

## STRUCTURES OF DE NOVO PROTEINS

One of the most exciting questions in protein biology regarding de novo genes is related to the structures of de novo proteins. Structural analyses indicate that many de novo proteins tend to exhibit disordered secondary structures, though the underlying reasons for this are debated (19, 54, 157). Three factors may contribute to this pattern. First, the functionality of a novel protein might necessitate flexible structures. Second, disordered structures could be less prone to forming harmful aggregates in cells and thus less likely to be purged (4, 70). Third, this disorder might emerge as a byproduct of GC content preferences of de novo genes (7). Future research is needed to dissect possibilities using large-scale functional methodologies or deep learning techniques.

A minority of de novo gene proteins may exhibit ordered structures. An attempt to investigate the structure of naturally evolving de novo genes involved biochemical and biophysical methods such as circular dichroism (CD), revealing that the BSC4 protein (16) forms soluble oligomers with a β-sheet secondary structure, a hydrophobic core, and a partially folded state (15). Further studies employing nuclear magnetic resonance (NMR) and CD demonstrated that a putative de novo protein, Goddard, contains a large α-helix and is also partially disordered (74). Ancestral sequence reconstruction combined with structural modeling is a useful approach to study structural changes of proteins (74, 107, 144). Recent advances using AlphaFold2 and ESMFold suggest that proteins encoded by a small subset of de novo genes might be stably folded (107). Molecular dynamics analysis helps researchers understand protein structures and dynamics in more realistic conditions (74, 107). Intriguingly, well-folded de novo proteins may emerge with a preexisting folded structure rather than through gradual structural changes over a short period (74, 107, 126). This observation implies that structurally distinct de novo genes are subject to purifying selection to maintain protein structures or the lack of structures for functional purposes.

While protein structures should theoretically be inferable from their sequences (3), and artificial intelligence methods are rapidly advancing (62), the extent to which de novo proteins can fold correctly in vivo remains almost completely unknown. Small proteins, such as those smaller than 100 amino acids, may fold spontaneously (143). However, larger proteins typically require chaperones for proper folding (143). Currently, there are no in-depth studies on how de novo proteins fold in vivo, nor are there any gene structures obtained from structural biological methods such as crystallography. This is a crucial area of study, as correct folding is vital for many functions, and misfolded proteins, prone to aggregation, can have deleterious effects on cells. Understanding the folding process of large de novo proteins and their avoidance of aggregation could offer significant insights into the potential and constraints of large de novo protein origination.

## DELETERIOUS DE NOVO PROTEINS AND HUMAN DISEASES

Most strongly deleterious novel ORFs and transcripts are likely to be quickly eliminated by purifying selection, making it challenging to identify such deleterious de novo ORFs in natural populations, even for model species for which population genome and phenotypic data are relatively abundant. However, the situation is different in human populations, where we have a better understanding of presumably deleterious phenotypes, such as diseases. It has long been recognized that new genes, such as chimeric genes created by rearrangement or translocation, may cause disease in humans (57). The role of de novo genes in human disease is poorly understood for multiple reasons, including that many are unannotated and that there is disagreement between studies about the numbers and criteria of human de novo genes (13). Nevertheless, Broeils et al. (13) manually curated the human de novo gene and disease literature and found five possible examples of de novo genes correlated with disease states, primarily cancer. Interestingly, among these genes were cases of ancestral lncRNAs that appear to have gained protein-coding capacity, specifically in humans. Thus, the genes are not novel, but corresponding proteins seem to be. Other cases appear to be bona fide novel genes, expressed only in humans or other primates. More generally, there is substantial interest in the possible role of the noncanonical proteome in human biology (110), including disease states. While most of the noncanonical proteome appears to derive from the aberrant translation of ancient genes, some transcripts that appear only in human disease may be de novo. Nevertheless, it remains poorly understood whether transcripts or proteins observed exclusively in human diseases are the causes or consequences of these disease states, necessitating further investigation.

## BENEFICIAL AND DELETERIOUS DE NOVO GENES AND THE ADAPTIVE IMMUNE SYSTEM

The immune system may be a hotspot for the origination of new genes (75) because it experiences strong selection from rapidly changing and diverse pathogens. Indeed, there are a few examples of putative de novo origins of immune-related genes, particularly antimicrobial peptides (14, 107). However, much less is known about how de novo genes contribute to the adaptive immune system (10). An intriguing research direction is to understand how novel proteins are presented by major histocompatibility complex (MHC) receptors on the cell surface and recognized as self. If identified as foreign, these novel deleterious ORFs could potentially cause autoimmune reactions (10). Tissue specificity and thymus expression might be expected outcomes for such novelties (10). A recent study has shown that there are thousands of translated, unannotated ORFs in the MHC-I-presented peptides in tumor samples, some of which might be species-specific ORFs (103). Understanding how beneficial novel ORFs become antigens and trigger immune responses while deleterious novel ORFs cause autoimmune diseases is important for future study.

## INSIGHTS FROM EXPERIMENTAL EVOLUTION AND RANDOMLY GENERATED SEQUENCES

One direction to investigate how new genes can be integrated into functional networks is to characterize randomly generated proteins or regulatory sequences. Naturally occurring de novo genes are not random, as the sequence compositions of nongenic regions in a genome are not random, and the de novo genes we observe have been filtered from a larger subset by natural selection. Nevertheless, studying randomly generated amino acid sequences or regulatory sequences offers insights into the potential functionality of entirely new sequences under specific artificial selective pressures. For example, random sequences may function as promoters (168) or enhancers (43). Studying the biochemical and biophysical properties of random sequences may bring novel insight into their cellular properties. Recent work has shown that random proteins can form secondary structures and are tolerated in vivo (142), and some random proteins are more soluble with the help of chaperones (54).

Random peptides may also provide a function in vivo. For instance, random peptides can exhibit a high binding affinity to streptavidin, particularly those sequences that include a short HPQ motif (159). Random proteins driven by a moderately strong promoter can positively affect the growth of *Escherichia coli* (96). While the proportion of beneficial random peptides in this study (96) might be overestimated due to concerns about proper null control (65, 154), this type of approach offers a novel perspective on whether and how completely new or foreign sequences can be functional (11). Random hydrophobic proteins may confer antibiotic resistance to *E. coli* (66, 67), enhance growth (32), or rescue knockout phenotypes (6, 41). These random proteins may operate through a number of distinct mechanisms, including binding to chaperones (41), interacting with untranslated regions (UTRs) (6), and directly functioning within the membrane (66, 67).

## DE NOVO GENE LOSS

Gene gain without loss would lead to excessively large genomes and gene numbers. Therefore, understanding gene loss in genome evolution is also important. Other types of genetic novelties, such as duplicated genes (87), microRNAs (miRNAs) (85), and orphan genes (105), often undergo frequent gains and losses. This pattern is also true for de novo genes (98, 125), including fixed genes (56) and segregating ones (99, 172). Similar to conserved genes (45, 112), the relative importance of selection and drift in gene loss is poorly understood (172). De novo transcripts have a high rate of turnover at the population level (49, 99, 119). While there are a few cases where conserved gene loss may be adaptive (91, 102, 141), it seems more likely that the turnover of young de novo genes reflects their loss by drift as the selective environment changes over time. Investigating the process and forces underlying the high turnover rate of the birth and death of functional de novo genes is an important goal.

## SMALL OPEN READING FRAMES/MICROPEPTIDES

In the vast genomic landscape, small proteins or micropeptides—typically fewer than 50 or 100 amino acids—have often been overlooked (131). Traditional gene prediction methods heavily rely on ORFs of a certain length and inadvertently filter out potential micropeptides. For example, only 125 out of 13,986 annotated proteins (FlyBase version 6.44, http://flybase.org; see also 104) are shorter than 50 amino acids in *D. melanogaster*. However, many more may be unannotated (28, 73). While there are some functional studies on a number of small peptides (121), most are unannotated or less studied, and many are still considered to be translational noise. However, the perception of small ORFs (smORFs) has shifted, especially after studies done on highly conserved examples. For instance, the smORF *mlpt* in *Tribolium* (124) and its homolog *tarsal-less* (11–32 amino acids long) in fruit flies (42, 69, 111) are crucial for development.

In the past few decades, technical developments in ribosome profiling (5, 58), improved mass spectrometry (128, 174), and bioinformatic tools (61, 88) have allowed for the capture and analysis of small proteins with increasing accuracy (94). Of these, ribosome profiling, which involves deep sequencing of ribosome-protected mRNA fragments to reveal actively translated genome regions, has been particularly impactful. This technique, not limited by ORF length, has uncovered many previously unrecognized micropeptides (5, 59, 150, 158).

An intriguing aspect of micropeptides is their possible contribution to de novo gene origination due to their shorter length (147, 171). Many identified micropeptides emerge from regions previously annotated as noncoding (2, 28), supporting a potential transition from nonprotein-coding sequences to functional coding regions through the formation of small genes. Most functionally well-known microproteins are evolutionarily conserved (2, 34, 42, 69). However, a few studies have revealed the possible roles of novel microproteins (28, 116, 147). Indeed, some well-studied de novo genes are shorter than (or slightly longer than) 100 amino acids, which, by definition, could be categorized as microproteins (1, 171).

The relationship between micropeptides and de novo genes is just beginning to be explored, presenting many unanswered questions. For instance, are the majority of de novo micropeptides identified by ribosome profiling functional? Traditional approaches for detecting evidence of selection, such as dN/dS, are underpowered for small proteins. Functional analysis is challenging and also requires careful work to confirm that the functions of a putative novel micropeptide are not attributable to lncRNAs, which adds further complexity to the analysis. More broadly, it is intriguing to hypothesize whether certain micropeptides follow a different evolutionary trajectory from other kinds of de novo genes. For example, do smORFs have a higher birth and death rate, making them more dynamic? Are smORFs more likely to be fixed through neutral processes than longer novel genes? Are they more abundant because more are likely to be nonfunctional noise? Large-scale and deep functional analyses are needed to help us understand the dynamics and function of de novo micropeptides.

## Figures and Tables

**Table 1:** Methods for detecting selection on de novo genes

Method to detect selection	Best used for	Advantage	Major limitation(s)
dN/dS-related methods	De novo genes fixed in two or more species	Can be calculated with PAML and other software	Ineffective on short timescales; not applicable to de novo lncRNAs; require a substantial number of substitutions to be meaningful
McDonald-Kreitman test	De novo genes fixed in two or more species	Overall more powerful than dN/dS	Requires population genomic data; not applicable to lncRNAs; requires a substantial number of substitutions to be meaningful
pN/pS	Polymorphic or species-specific genes	Can be measured relatively easily	Requires population genomic data; power is unclear for short genes; requires a substantial number of substitutions to be meaningful
Footprints of genetic hitchhiking	Can be used on any gene or on species-specific genes varying in frequency	Can be applied to coding and noncoding genes	Require population genomic data; susceptible to confounding by population history; problematic in low-recombination or clonal organisms
Candidate frequency correlation with transcript properties	Species-specific candidate genes	Inferences made based on assumed properties of transcript length and expression level	Assumptions need validation; conclusions are stronger when supported by additional evidence
HKA and HKA-like statistics	Any candidate gene	Applicable to any candidate gene	Dependent on sequence information in both the focal population and outgroup species; problematic in low-recombination or clonal organisms

## SUMMARY POINTS

1. De novo gene origination is a mechanism for the origination of new genes and has been described in many taxa.
2. Investigation of de novo genes provides a unique opportunity to study the origin of gene expression regulation, the effect of selection and neutral processes in gene evolution, and the origin and function of de novo proteins.
3. Despite increasing numbers of studies, robust identification of de novo genes remains challenging. There is considerable variation among studies in estimates of de novo gene abundance, even in model systems. Improved methods are urgently needed.
4. Positive selection and adaptation may play an important role in the fixation and maintenance of de novo genes in some taxa. Genomic conflicts and host–pathogen interactions may be common sources of natural selection favoring the spread of de novo genes. Neutral processes may also play a role in some species.
5. De novo genes may impact human health, including in areas such as cancers, rare diseases, and precision medicine.

## FUTURE ISSUES

1. What are the functions of de novo genes, and how do they integrate into functional networks?
2. To what extent do de novo genes function as noncoding RNAs? What are the functions of de novo long noncoding RNAs, and how do they function?
3. What factors determine de novo gene persistence time? What roles do genomic conflicts and host–pathogen interactions play in de novo gene evolution?
4. What is the minimal structure or protein sequence length for de novo protein function? How do disordered de novo proteins function?
5. What new methods, including machine learning techniques, can be applied in population and comparative genomics to identify de novo genes and infer the role of natural selection, especially in nonmodel species?
6. Do long de novo genes and small open reading frames have different evolutionary paths and functional consequences?
7. Why are de novo genes predominantly enriched in the testis in animal species? What are the relative roles of mutation and natural selection in de novo gene origination, and how do these factors vary across functions and cell types?
8. How are vertebrate de novo antigens presented as self, and how do they avoid triggering autoimmune responses?
